# Biodistribution of ^131^I in mice is influenced by circadian variations

**DOI:** 10.1038/s41598-020-72180-7

**Published:** 2020-09-23

**Authors:** Charlotte K. Andersson, Mikael Elvborn, Johan K. E. Spetz, Britta Langen, Eva B. Forssell-Aronsson

**Affiliations:** 1 Department of Radiation Physics, Inst of Clinical Sciences, Sahlgrenska Cancer Center, Sahlgrenska Academy at the University of Gothenburg, Sahlgrenska University Hospital, SE 413 45 Gothenburg, Sweden; 2 Department of Medical Physics and Biomedical Engineering, Sahlgrenska University Hospital, SE 413 45 Gothenburg, Sweden

**Keywords:** Thyroid diseases, Radiotherapy

## Abstract

Effects of radiation and biodistribution of radionuclides are often studied in animal models. Circadian rhythm affects many biological functions and may influence the biokinetics of radionuclides and observed responses. The aim of this study was to investigate if the time during the day of ^131^I injection affects the biodistribution and absorbed dose to tissues in mice. Biodistribution studies were conducted on male C57BL/6 N mice for three diurnal time-series: the animals were i.v. injected with 160 kBq ^131^I at 8 am, 12 pm or 4 pm. The activity concentration in organs and tissues was measured at 1 h to 7 days after administration and absorbed dose at day 7 was determined. Comparison between the three time-series showed statistically significant differences in activity concentration in all investigated tissues and organs. Administration performed at 12 pm resulted in general in higher absorbed dose to the organs than injection performed at 8 am and 4 pm. Time of day of administration affects the biodistribution of ^131^I in mice and consequently the absorbed dose to individual organs. These findings advocate that subsequent biodistribution studies and dosimetry calculations should consider time-point of administration as a variable that could influence the results.

## Introduction

^131^I is a well-established radionuclide used in treatment of various thyroid disorders, such as hyperthyroidism and differentiated thyroid cancer. Even if the uptake of iodine is very specific to thyroid tissue, side effects from off-target accumulation are common. Frequent short-term side effects after ^131^I therapy of patients with differentiated thyroid cancer are gastrointestinal symptoms, pain or swelling in the neck or salivary glands, while frequent late effects are functional problems with salivary glands^1–5^. Reliable dosimetry, based on detailed knowledge of radionuclide biodistribution/biokinetics, is needed to determine potential risks from radionuclide therapy.

Many biological functions in living organisms are regulated in an oscillating manner. Heart rate, sleep/wake cycle, and body temperature, as well as secretion of hormones like thyroid stimulating hormone (TSH), insulin, and melatonin are but a few examples of processes that follow a circadian rhythm^6^. These time-related processes are regulated by a system of endogenous biological clocks, i.e. a time-keeping network^7^. In mammals, the suprachiasmatic nucleus (SCN) is the master clock and major pacemaker. SCN is located in the hypothalamus and is regulated by stimuli from the environment such as light, social activity and food intake^6^. Through hormones and the neuronal system, the SCN in turn synchronizes the timing of oscillators in peripheral organs, where the hypothalamus-pituitary-thyroid (HPT) axis is an example of an endocrine feedback loop that is known to have a circadian rhythm^8^.

Although circadian rhythm is known to be important for a number of responses, e.g. expression of radiation-induced apoptosis^9,10^, studies on its application in the field of radionuclide therapy are quite limited^11^. Only a few studies have been performed to address diurnal variations in uptake of ^131^I. Walinder demonstrated already in 1971 that the accumulated activity of ^131^I in the thyroid in mice has a diurnal variation^12^. Such effects have also been observed in male rat^13,14^. However, these studies focused only on the thyroid and did not investigate diurnal variations in the biodistribution of ^131^I. We recently reported that both the intensity and biological function of transcriptomic responses to i.v. administered ^131^I vary with the time of day of administration^15^. To the best of our knowledge, no study has been published on the question whether the biodistribution/biokinetics of ^131^I also exhibits diurnal variations. The purpose of this study was to investigate if time of day of injection affects the biodistribution/biokinetics of ^131^I and the absorbed dose to organs and tissues in mice.

## Materials and methods

### Animal model

Animals used were 9–10 weeks old male C57BL/6 N mice (Charles River Laboratories International, Inc., Sulzfeld, Germany) weighing 24 g (SD = 2 g). The mice were kept under standard laboratory day and night cycle, i.e. darkness from 6 pm to 6 am. Water and laboratory food with iodine concentration of 0.87 µg/g were given ad libitum. The study was approved by the Ethics Committee for Animal Research in Gothenburg (no. 146–2015). All experiments were performed in accordance with relevant guidelines and regulations.

### ^131^I administration and organ sampling

Altogether 165 animals were divided into three main groups and were i.v. injected with 160 kBq ^131^I (GE Healthcare, Braunschweig, Germany) in physiological saline solution (0.1 ml) in the tail vein at 8 am, 12 pm or 4 pm, respectively. The animals were killed by cardiac puncture under anesthesia with sodium pentobarbital (APL, Sweden) after 1, 4, 8, 18, 24, 72, or 168 h following injection (n = 5 – 10 per subgroup).

The thyroid, salivary glands, lungs, heart, spleen, liver, kidneys, and stomach were excised and blood and stomach contents were sampled. Several millimeters of the large intestine (starting from the sigmoid colon) and of the small intestine (starting from the duodenum) were excised and contents from the large and small intestine were collected. Sample weight and radioactivity content were measured directly after excision. The average thyroid weight of 46 collected thyroids (weighing ca 3 – 5 mg) was 3.9 mg (SEM = 0.1), a value well in accordance with literature^12^. A selection of thyroids was fixed in formalin immediately after weighing; 35, 30 and 30 samples for injection at 8 am, 12 pm and 4 pm, respectively.

### Radioactivity measurements

The ^131^I activity in the stock solutions was measured using a CRC-15 dose calibrator ion chamber (Capintec, IA, USA) and the activity concentration determined. For each animal, the syringe was weighed before and after injection. To compensate for adsorbed ^131^I inside the syringes, control syringes were used to determine the actual activity of the injected solution. The ^131^I content of the control syringes was measured using a Wallac 1480 Wizard® 3″ NaI(Tl) gamma counter (Wallac Oy, Turku, Finland).

^131^I activity in tissue samples was measured in the gamma counter. Corrections were performed for background and dead time loss. Self-attenuation of ^131^I in the sample and geometric effects were investigated and found negligible. All data were decay corrected to time of administration.

The activity concentration in organs and tissues at different times after injection, $c_{tissue} \left( t \right)$, was calculated as percent of injected activity per organ mass (%IA/g):

1 $$c_{tissue} \left( t \right) = \frac{{A_{tissue} \left( t \right)}}{{A_{inj} \cdot m_{tissue} }} \cdot 100\% ,$$

where $A_{tissue} \left( t \right)$ is the activity in the sample at time *t* corrected for radioactive decay to time of administration (*t* = 0), $A_{inj}$ is the injected activity at time *t* = 0, and $m_{tissue}$ is the mass of the sample. Due to the uncertainties in the thyroid mass measurements, the ^131^I activity in the thyroid is presented as percent of injected activity (%IA).

### Histological evaluation

Altogether, 37 thyroid samples with low ^131^I activity concentration (less than 20% of group max) were analyzed. The samples were fixed in formalin immediately after weighing and then embedded in paraffin, sectioned in 4 μm slices and stained with haematoxylin–eosin, according to standard protocols. Thyroid tissue samples containing less than approximately 25% thyroid tissue were excluded from the study.

### Absorbed dose calculation

The absorbed dose from ^131^I was calculated according to the MIRD formalism^16^:

2 $$D\left( {r_{T} ,T_{D} } \right) = \mathop \sum \limits_{{r_{S} }} \frac{{\tilde{A}\left( {r_{S} ,T_{D} } \right)}}{{M\left( {r_{T} } \right)}}\mathop \sum \limits_{i} E_{i} Y_{i} \varphi (r_{S} \leftarrow r_{T} ),$$

where $\tilde{A}\left( {r_{S} ,T_{D} } \right)$ is the time-integrated activity over the dose-integrated period $T_{D}$ in the source organ $r_{S}$ and $M\left( {r_{T} } \right)$ is the mass of the target organ $r_{T}$. $Y_{i}$ is the yield of radiation $i$ with energy $E_{i}$ and $\varphi \left( {r_{S} \leftarrow r_{T} } \right)$ is the fraction of the radiation in the source organ that is absorbed in the target organ^16^. For thyroid, the average weight (3.9 mg, SD = 0.5) was used. The calculations were based on assumption of homogeneous activity distribution in the organs.

Only the electron emission was considered in the absorbed dose calculations, and therefore $E_{i} Y_{i}$ was set to 191 keV/Bqs^17^. The self-absorbed fraction for blood was set to 1. For the salivary glands the self-absorbed fraction was estimated to be 0.880 by interpolation of data for absorbed fraction and tissue weight for organs of similar weight, i.e. lungs and stomach. The cross-absorbed fractions from the salivary glands to the other organs was set to 0. The absorbed fractions for the other investigated organs were taken from a mouse model matching our study design^18^.

The time-integrated activity was estimated with the trapezoidal rule from the time of administration to the dose determination time based on the data from the biodistribution. The activity in the blood at t = 0 was assumed to be equal to the injected activity. For the rest of the organs and tissues the activity at t = 0 were set to zero.

The relative difference between the absorbed dose to the organs in two groups with different time of day of ^131^I injection (groups 1 and 2) were calculated:

3 $$\frac{{\left| {D_{1} - D_{2} } \right|}}{{\frac{{D_{1} + D_{2} }}{2}}}$$

### Statistical analyses

ANOVA with Tukey HSD test was used to determine the statistical significance of differences in activity concentration between groups. For the thyroid, the statistical significance of differences in ^131^I activity was determined by Kruskal–Wallis one-way ANOVA with pairwise comparison, using IBM SPSS Statistics for Windows 25.0. Statistical significance was considered for probabilities higher than 95% (p < 0.05). Uncertainties in the measurements are given as the standard error of the mean (SEM).

## Results

### Biodistribution of ^131^I

**Figure 1:**
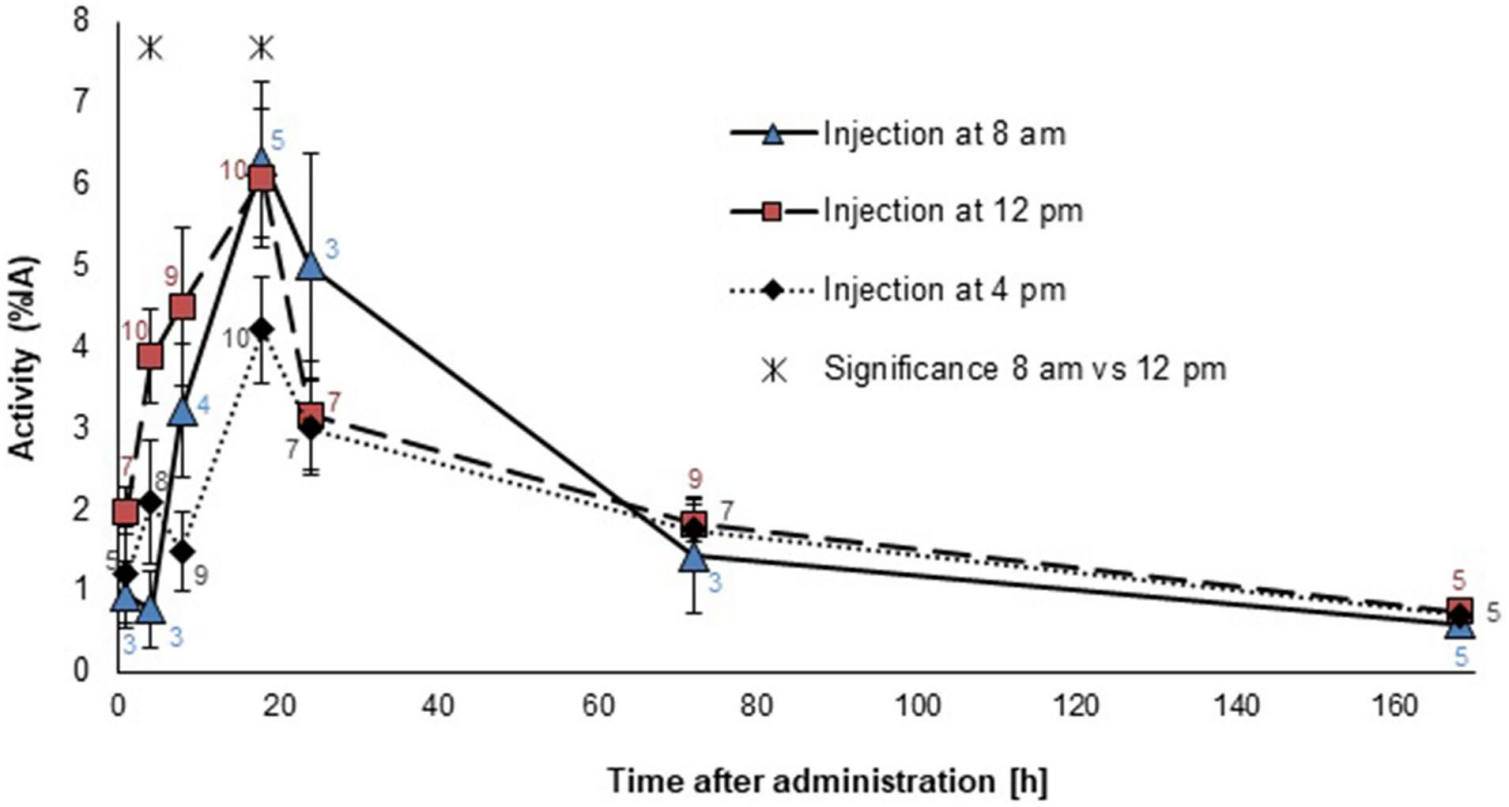
^131^I activity in the thyroid given as percent of injected activity from 1 h to 7 days after administration of 160 kBq ^131^I at 8 am, 12 pm and 4 pm. Data labels represent the number of animals and vertical error bars indicate SEM. Statistically significant differences between 8 am and 12 pm are noted by stars.

The biodistribution of ^131^I was determined for 1 h up to 7 days post injection (p.i.) performed at 8 am, 12 pm or 4 pm. The ^131^I activity in the thyroid (%IA) is presented in Fig. 1. Highest median value was observed at 18 h p.i. for all administration time-points; 6.3 (SEM = 1.0) for 8 am, 6.1 (SEM = 0.9) for 12 pm and 4.2 (SEM = 0.7) for 4 pm. Maximum for the 4 pm group was statistically significant lower than for the 8 am group (p = 0.046). Statistically significant differences were also observed between the 8 am and 12 pm groups at 4 h p.i., 0.77 (SEM = 0.47) vs. 3.9 (SEM = 0.6), respectively (p = 0.018). A total of 37 thyroid samples had unexpectedly low ^131^I activity and 31 of were excluded from the study due to low thyroid tissue content based on histological analysis.

The ^131^I concentration in the other investigated tissues is presented in Table 1. High ^131^I activity concentration was found in stomach content, stomach, and salivary glands. In total, statistically significant differences between the administration time-points were observed in 38 out of 91 time-points and samples. For most investigated organs and tissues, the maximum activity concentration was reached at the first time-point (1 h p.i.) followed by an exponential decrease. For injection performed at 4 pm, the decrease was generally more rapid and the decrease was less rapid for injection performed at 12 pm. The activity concentration in the kidneys did not depict a monotonic decline: a local maximum was observed at 3 days.

**Table 1:** The activity concentration in mouse tissues at 1 h to 7 days after injection of 160 kBq ^131^I at three injection times (8 am, 12 pm and 4 pm).

		^131^I activity concentration (%IA/g)						
Tissue	Injection time	1 h	4 h	8 h	18 h	24 h	3 days	7 days
Blood	8 am	4.3 *0.5*	1.2^†^ *0.2*	0.78 *0.07*	0.14^†,‡^ *0.01*	0.057 *0.007*	0.056 *0.003*	0.020 *0.002*
	12 pm	4.9 *0.3*	2.0* *0.1*	1.2^‡^ *0.3*	0.080* *0.007*	0.075 *0.008*	0.057 *0.009*	0.015 *0.001*
	4 pm	5.4 *0.7*	1.0 *0.1*	0.32^†^ *0.08*	0.058* *0.007*	0.066 *0.008*	0.046 *0.007*	0.21 *0.17*
Heart	8 am	1.8 *0.1*	0.42^†^ *0.05*	0.31 *0.03*	0.075^†,‡^ *0.003*	0.038 *0.003*	0.031 *0.002*	0.012 *0.002*
	12 pm	2.0 *0.1*	0.9* *0.1*	0.47 *0.12*	0.052* *0.005*	0.049 *0.006*	0.033 *0.003*	0.0096 *0.000*
	4 pm	2.1 *0.2*	0.43 *0.04*	0.16 *0.03*	0.037* *0.004*	0.048 *0.009*	0.027 *0.003*	0.026 *0.011*
Kidneys	8 am	3.0 *0.2*	0.74^†^ *0.09*	0.48 *0.04*	0.17 *0.01*	0.26 *0.04*	1.4 *0.1*	0.79 *0.16*
	12 pm	3.0 *0.1*	1.5* *0.1*	0.68^3^ *0.15*	0.17 *0.02*	0.27 *0.03*	1.2 *0.1*	0.64 *0.05*
	4 pm	3.3 *0.3*	0.73 *0.06*	0.19^†^ *0.04*	0.14 *0.03*	0.24 *0.03*	0.89 *0.15*	0.55 *0.09*
Large intestine	8 am	2.6 *0.2*	0.83^†^ *0.08*	0.58 *0.04*	0.12^†,‡^ *0.02*	0.049 *0.007*	0.033 *0.002*	0.014 *0.003*
	12 pm	3.2 *0.2*	1.5* *0.1*	0.70^‡^ *0.14*	0.072* *0.007*	0.072 *0.012*	0.041 *0.003*	0.017 *0.002*
	4 pm	3.3 *0.5*	0.81 *0.09*	0.21^†^ *0.05*	0.052* *0.007*	0.08 *0.014*	0.040 *0.004*	0.28 *0.13*
Large intestine contents	8 am	2.8 *0.7*	3.5 *0.6*	3.0 *0.4*	0.45^†,‡^ *0.07*	0.23 *0.03*	0.16 *0.03*	0.060 *0.004*
	12 pm	2.3 *0.3*	5.4 *0.6*	4.5^‡^ *1.2*	0.24* *0.02*	0.19 *0.01*	0.084^‡^ *0.016*	0.10 *0.03*
	4 pm	3.0 *0.5*	3.2 *0.6*	0.76^†^ *0.21*	0.16* *0.02*	0.24 *0.04*	0.26^†^ *0.06*	0.095 *0.008*
Liver	8 am	1.7 *0.1*	0.45^†^ *0.06*	0.34 *003*	0.12^‡^ *0.00*	0.098 *0.010*	0.10 *0.01*	0.035 *0.003*
	12 pm	1.9 *0.1*	0.85* *0.07*	0.48^‡^ *0.10*	0.10 *0.01*	0.11 *0.01*	0.10 *0.01*	0.032 *0.001*
	4 pm	2.1 *0.2*	0.43 *0.04*	0.15^†^ *0.03*	0.072*^,†^ *0.007*	0.093 *0.009*	0.089 *0.011*	0.084 *0.035*
Lungs	8 am	3.9 *0.3*	1.0^†^ *0.1*	0.67 *0.05*	0.16^‡^ *0.01*	0.077 *0.006*	0.062 *0.004*	0.023 *0.002*
	12 pm	4.4 *0.2*	2.0* *0.1*	0.93^‡^ *0.18*	0.12^‡^ *0.01*	0.11 *0.01*	0.068 *0.007*	0.024 *0.001*
	4 pm	4.8 *0.6*	0.95 *0.08*	0.28^†^ *0.06*	0.081*^,†^ *0.005*	0.088 *0.011*	0.054 *0.006*	0.10 *0.04*
Salivary glands	8 am	12 *2*	6.4^†^ *1.1*	4.1^‡^ *0.6*	0.79^†,‡^ *0.06*	0.18 *0.02*	0.065 *0.005*	0.022 *0.003*
	12 pm	12 *1*	12* *1*	4.8^‡^ *0.8*	0.35*^,‡^ *0.05*	0.19 *0.02*	0.092 *0.012*	0.026 *0.003*
	4 pm	11 *2*	5.1 *0.6*	1.5*^,†^ *0.4*	0.17*^,†^ *0.02*	0.19 *0.01*	0.066 *0.008*	0.12 *0.06*
Small intestine	8 am	2.9 *0.2*	0.98 *0.06*	0.67 *0.1*	0.25^†,‡^ *0.01*	0.087 *0.003*	0.048 *0.003*	0.0021^‡^ *0.001*
	12 pm	3.8 *0.4*	1.7 *0.2*	1.2^‡^ *0.2*	0.16*^,‡^ *0.02*	0.088 *0.011*	0.06 *0.005*	0.019^‡^ *0.002*
	4 pm	3.6 *0.4*	1.0 *0.2*	0.37^†^ *0.07*	0.098*^,†^ *0.014*	0.084 *0.012*	0.053 *0.006*	0.18*^,†^ *0.06*
Small intestine contents	8 am	5.7 *0.5*	1.8 *0.3*	1.8 *0.3*	0.30^‡^ *0.02*	0.14 *0.01*	0.12 *0.01*	0.035 *0.004*
	12 pm	11 *5*	4.7 *1.2*	1.9 *0.6*	0.24^‡^ *0.03*	0.15 *0.02*	0.16 *0.03*	0.055 *0.012*
	4 pm	5.7 *0.4*	1.4 *0.2*	0.40 *0.10*	0.13*^,†^ *0.02*	0.14 *0.03*	0.14 *0.02*	0.50 *0.36*
Spleen	8 am	2.7 *0.2*	0.72^†^ *0.11*	0.45 *0.02*	0.10^†,‡^ *0.00*	0.056 *0.006*	0.031 *0.002*	0.011 *0.001*
	12 pm	3.0 *0.1*	1.4* *0.1*	0.66^‡^ *0.13*	0.070*^,‡^ *0.006*	0.051 *0.003*	0.033 *0.005*	0.011 *0.001*
	4 pm	3.1 *0.3*	0.63 *0.06*	0.20^†^ *0.04*	0.046*^,†^ *0.004*	0.050 *0.004*	0.030 *0.004*	0.046 *0.023*
Stomach	8 am	28 *5*	7.7^†^ *1.7*	5.1 *0.7*	0.86^‡^ *0.07*	0.21^‡^ *0.03*	0.071 *0.007*	0.024^‡^ *0.002*
	12 pm	36 *4*	13* *1*	6.2^‡^ *1.5*	0.6 *0.12*	0.33 *0.03*	0.22 *0.1*	0.046^‡^ *0.002*
	4 pm	38 *4*	6.7 *1.0*	1.3^†^ *0.3*	0.28* *0.03*	0.37* *0.05*	0.12 *0.02*	0.11*^,†^ *0.025*
Stomach contents	8 am	78 *10*	21^†^ *3*	14 *2*	1.6 *0.3*	0.4 *0.08*	0.11^‡^ *0.02*	0.048^‡^ *0.010*
	12 pm	110 *20*	37* *4*	18^‡^ *5*	1.8^‡^ *0.6*	0.74 *0.08*	0.32 *0.05*	0.15 *0.05*
	4 pm	180 *42*	17 *3*	3.2^†^ *0.8*	0.51^†^ *0.08*	1.4 *0.6*	0.58* *0.18*	0.2* *0.04*

Data are presented as mean (n = 4–10) and SEM (italics). *, ^†^, ^‡^ indicate statistically significant differences from injection at *8am, ^†^12 pm and ^‡^4 pm, respectively.

### Dosimetry

**Figure 2:**
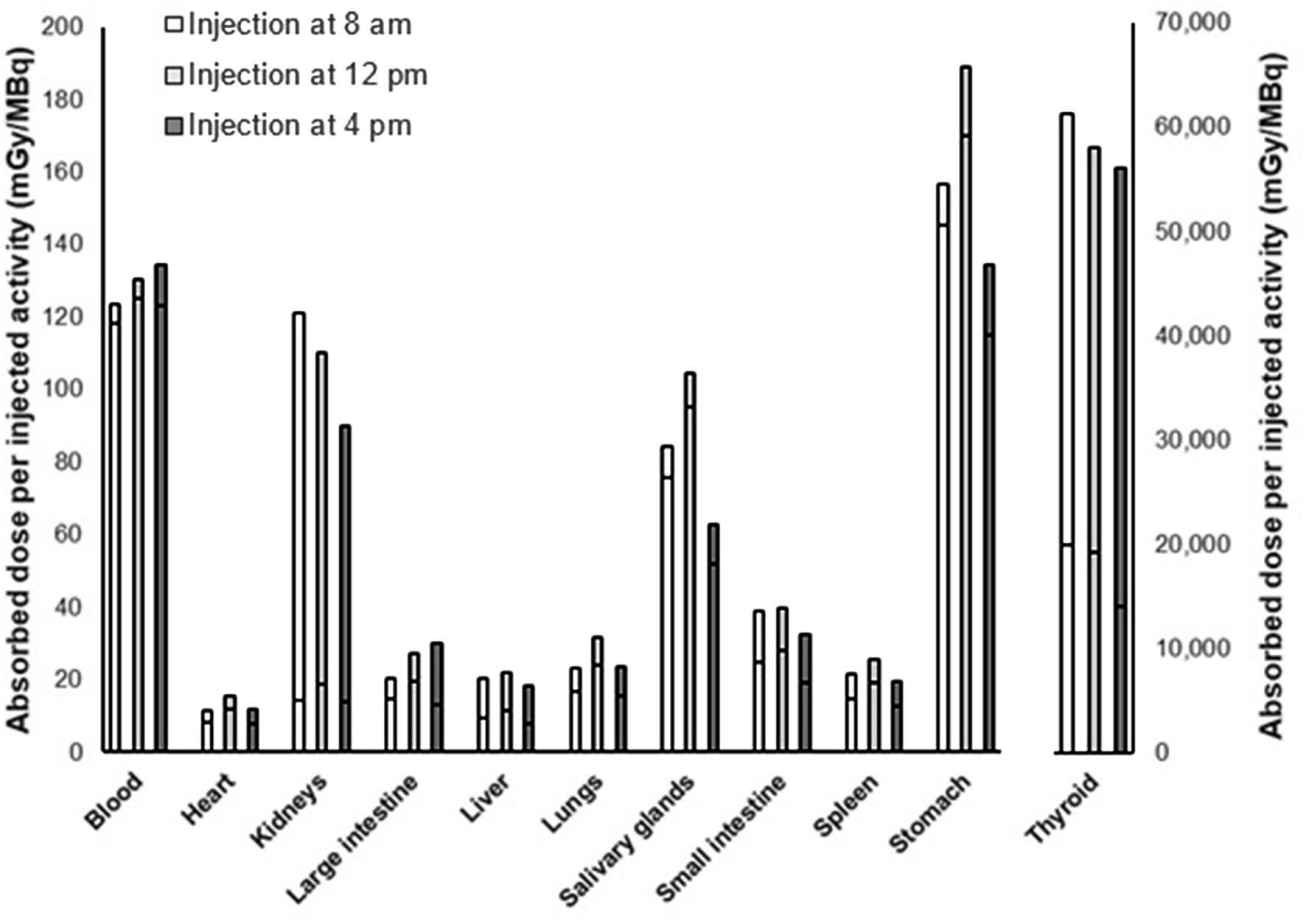
Absorbed dose to organs per injected activity after injection of 160 kBq ^131^I at 8 am, 12 pm and 4 pm. Bottom segment of each bar indicates absorbed dose per injected activity after 24 h and total bar indicates absorbed dose per injected activity after 7 days.

Absorbed doses to organs per injected ^131^I activity are presented in Fig. 2. The thyroid received the highest absorbed dose: 61 Gy/MBq (8 am), 58 Gy/MBq (12 pm), and 56 Gy/MBq (4 pm) at 7 d after injection. The stomach, blood, kidneys and salivary glands received lower absorbed doses than the thyroid, but still higher than the other organs investigated. The relative difference in absorbed dose between the experiments was highest for the salivary glands with up to 50% relative difference (100 mGy/MBq, 12 pm *vs*. 63 mGy/MBq, 4 pm). Other organs with a distinct difference are for example stomach (34%, 190 mGy/MBq, 12 pm *vs.* 130 mGy/MBq, 4 pm) and large intestine (38%, 20 mGy/MBq, 8 am *vs.* 30 mGy/MBq, 4 pm).

## Discussion

Diurnal variations in biodistribution of ^131^I in mice were investigated by performing similar biodistribution studies with different time of administration. Statistically significant differences were observed at least at one time-point in all investigated tissues and organs, most commonly at 4, 8 and 18 h p.i. The differences in activity concentration may be a consequence of differences in physiological processes as a result of circadian rhythm.

When investigating biodistribution of radioiodine, the thyroid is the primary organ of interest. In this study, we demonstrated a difference in uptake of ^131^I in the thyroid during the first day. Maximum was reached at the same time-point in all three studies, but there was a 33% difference in maximum activity between the 8 am and 4 pm groups. These findings are in agreement with Walinder’s study on mouse resulting in lower thyroid uptake 24 h after injection at 4 pm compared with injection at 8 am or 1 pm^12^. On the contrary, studies on rat showed that the ^131^I uptake in thyroid was higher if the injection was performed in the afternoon (7 pm or 6 pm) compared with injection in the morning (7 am or 8 am)^13,14^. The different diurnal patterns could be explained by the species difference and the much lower iodine concentration in the food (0.07 µg/g) given to the rats.

The circadian rhythm of the hypothalamus-pituitary-thyroid (HPT) axis may be an underlying cause of the diurnal variations in iodine uptake in the thyroid. The HPT axis regulates the secretion of T3, T4 and TSH^8^, resulting in oscillating levels of the hormones in the blood^19,20^. TSH increases the iodine uptake by stimulating iodide transport into the thyrocytes via the sodium/iodide symporter (NIS)^21–23^. It is therefore possible that variations in thyroidal uptake are caused by fluctuating TSH levels in the blood.

Most of the T3 is produced from conversion of T4 to T3 by the type 1 iodothyronine deiodinase (D1) in the liver, kidneys and thyroid^24^, where iodide is released from the hormone. This could explain the increase of ^131^I observed in the kidney after 24 h. The local maximum could be an effect of the decrease of ^131^I in the thyroid caused by excretion of T3 and T4 containing ^131^I. Since the kidneys store more T4, T3 and iodide per organ weight than the liver^25^ and due to its important role in excretion of iodide, it is possible that this effect is observable in the kidney and not in the liver.

In most organs the decrease in activity concentration (after maximum) was less steep for injection performed at 12 pm. This difference resulted in higher mean absorbed dose to most organs of the animals injected at 12 pm. The maximal relative difference in mean absorbed dose to the thyroid delivered during the first 7 days was 9%, depending on the time-point of administration, while greater differences were observed in other organs, such as the salivary glands and stomach. Unfortunately, no statistical methods can be applied to determine if these differences are statistically significant. Furthermore, the biological significance of 9% difference in absorbed dose to the thyroid is unknown and needs to be further investigated.

Large inter-individual differences in uptake of radioiodine in thyroid have been shown in previous animal studies^26,27^. Individual metabolic differences and food intake may partly explain the spread within an animal group, since intake of stable iodine via the food may reduce the uptake of radioiodide^12^. In this study the animals were given food ad libitum, in agreement with standard laboratory care.

In chronotherapy, administration of, *e.g.*, anti-cancer drugs is performed at specified time points of day in order to optimize the treatment^11^. Chronotherapy with cytotoxic drugs have shown promising results with variations of over 50% in efficiency dependent on time of day of administration^28^. A few studies have also investigated chronotherapy with external radiotherapy^29–31^ and it has been proposed that adaptation to the circadian rhythm can result in a more sufficient personalized treatment, especially for pediatric patients^32^. The number of studies on diurnal effects related to radiopharmaceuticals are even fewer. A handful of studies from the 50 s, 60 s and 70 s have observed circadian variations in ^32^P (orthophosphate) uptake in mammary tumors^11,33–35^. The intention then was to use ^32^P as a marker for mitotic activity in the tumors and thereby be able to choose a time-point for external radiotherapy when tumor is more radiosensitive. However, as far as we know, chronotherapy with radiopharmaceuticals is yet unexplored.

The findings in the present study suggest that ^131^I-based radionuclide therapy may be a potential candidate for chronotherapy. Interestingly, our data show that in mice about 30–50% lower absorbed dose to salivary glands, stomach and small intestines could be obtained if administration was made at 4 pm compared with at 12 pm. Administration at 4 pm also gave lower absorbed dose to thyroid (that might reflect tumor behavior) but to a lower extent, demonstrating that lower side effects could potentially be obtained in salivary glands and gastrointestinal tract if ^131^I was administered late during the day. Clinically, the relevance of a difference in absorbed dose to thyroid of 9% might be of minor importance if thyroid is the target and of greater importance if it is a risk organ. If biodistribution of ^131^I could be optimized with respect to time of administration also in humans, chronotherapy of patients with differentiated thyroid cancer could work as an additional therapeutic tool or method for improving quality of life.

In the present study animals were i.v. injected with ^131^I, while in the clinic, many patients with thyroid diseases receive ^131^I orally in pill or in liquid form. The difference in route of administration should not affect the scope of this study, since absorption of orally administered ^131^I to the blood is rapid and virtually complete^36,37^. Hence, i.v. injection of ^131^I can be seen as a simulation of oral administration. Furthermore, an equivalent patient study would have required several test doses for one and the same patient due to the large inter-individual differences in thyroid uptake. Repeated administrations of ^131^I, even with several months between, would pose a great risk of thyroid stunning which not only would affect the results of the study but also the treatment effect. From an ethical point of view, we therefore found it appropriate to investigate potential effects of circadian rhythm in mice before clinical studies are initiated. Another reason for animal studies was the possibility to obtain data also for non-thyroid tissues.

## Conclusion

The results of this study demonstrated differences in biodistribution and biokinetics and consequently differences in absorbed dose due to time of ^131^I administration. These findings suggest that diurnal variations should be considered in dosimetric evaluations of radiopharmaceuticals. Chronotherapy using ^131^I-based radiopharmaceuticals could be favorable for reduction of short- and long-term side effects, and should be further investigated.

## Data Availability

The data generated and analyzed during this study are available from the corresponding author upon reasonable request.
